# Predictors of Medication Adherence Among Taiwanese Community-Based Adults with Hypertension: The Application of Temporal Self-Regulation Theory

**DOI:** 10.3390/bs16071075

**Published:** 2026-07-01

**Authors:** Dibora Teferi Haile, Yueh-Ching Yu, Yen-Jung Chang

**Affiliations:** 1 Department of Health Promotion and Health Education, National Taiwan Normal University, Taipei City 106308, Taiwan; 81405008e@ntnu.edu.tw (D.T.H.); 61105006e@gapps.ntnu.edu.tw (Y.-C.Y.); 2 School of Public Health, College of Health Science and Medicine, Wolaita Sodo University, Soddo 4620, Ethiopia

**Keywords:** medication adherence, hypertension, temporal self-regulation theory, Taiwan, community-based

## Abstract

Medication non-adherence remains a persistent public health concern among individuals living with hypertension, where a gap between intention and actual behavior continues to undermine effective blood pressure control. Guided by temporal self-regulation theory (TST), this study examined the roles of intention, behavioral prepotency, and self-regulatory capacity in shaping medication adherence. A cross-sectional telephone survey was conducted in 2024 among a nationally representative sample of 1068 adults with hypertension in Taiwan. Data were collected on Adherence to Refills and Medications Scale (ARMS) adherence score, behavioral constructs, and sociodemographic and clinical characteristics using validated instruments, including the Adherence to Refills and Medications Scale, Behavioral Prepotency Questionnaire, and Brief Self-Control Scale. Multiple linear regression analyses were used to identify predictors and test the moderation effects. Participants had a mean age of 69.4 years, with 54.3% being male. Overall, the ARMS adherence score was moderate (mean = 15.89, SD = 3.53). TST-related constructs explained 36% of the variance in the ARMS score. Beyond behavioral intention, both habit strength and self-regulatory capacity emerged as significant predictors of medication adherence. However, neither construct significantly moderated the intention–adherence relationship. Additionally, older individuals and those with lower educational attainment demonstrated lower ARMS scores, indicating better adherence. These findings highlight the importance of habit formation and self-regulatory processes in promoting sustained medication adherence. These behavior-focused strategies may also inform management of other chronic conditions.

## 1. Introduction

Hypertension is one of the leading global causes of mortality and disability, currently affecting an estimated 1.4 billion people worldwide (World Health Organization, 2025; Yang et al., 2025), and its prevalence continues to rise rapidly each year (Egan et al., 2024). Beyond its high prevalence, hypertension is a major driver of cardiovascular diseases, substantially increasing the risk of heart disease, stroke, kidney failure, and diabetes-related blindness (World Health Organization, 2024). In 2021, hypertensive heart disease (HHD) alone affected approximately 12.5 million individuals globally, resulting in about 1.33 million deaths and nearly 25.5 million disability-adjusted life years (DALYs), highlighting the enormous burden it places on global health (Wu et al., 2025). According to the National Nutrition and Health Survey, the prevalence of hypertension in Taiwan is approximately 26.8% (Health Promotion Administration, 2023), and hypertension represented the fourth highest category of medical expenditure in 2022 (Health Promotion Administration, 2024).

Adherence is defined as the extent to which an individual’s behavior, including medication-taking, aligns with agreed recommendations from a healthcare provider. It is not merely compliance, but rather an active, voluntary, and collaborative involvement of the individual in a mutually agreed therapeutic plan to achieve optimal health outcomes (Ahmed & Aslani, 2014; Sabaté, 2003). Adherence to antihypertensive medication is crucial to control blood pressure (BP) and hypertension management outcomes (Choudhry et al., 2022). Individuals with moderate to good medication adherence levels had a notably lower risk of uncontrolled blood pressure compared to those with low adherence (Hossain et al., 2025) and a reduced risk of complications (Feng et al., 2022). However, many individuals receiving treatment forget to take their medications or run out of them within a year (Gadkari & McHorney, 2012). Among individuals with hypertension, suboptimal medication adherence has been independently linked to poorer clinical outcomes (Kim et al., 2025) and a higher risk of cardiovascular disease (CVD) events and mortality (Rodriguez et al., 2019). In recent studies conducted in Taiwan, about one fourth (25.8%) of participants were non-adherent to prescribed medications (Lin et al., 2024), and non-adherence to antihypertensive therapy has been linked to higher hospitalization rates (Chou et al., 2020). Medication non-adherence remains a critical challenge in hypertension management, highlighting the urgent need for effective adherence strategies (Gadkari & McHorney, 2012; Piña et al., 2021).

### Temporal Self-Regulatory Theory

The Theory of Planned Behavior (TPB) is widely applied to explain antihypertensive medication adherence (Ajzen, 1991; Bane et al., 2006; Liddelow et al., 2020b). While the TPB highlights intention as a key predictor of behavior, it fails to fully explain the gap between intention and adherence. Substantial evidence demonstrates an “intention–behavior gap,” whereby even strong motivation leads to only modest changes in actual behavior (Webb & Sheeran, 2006). In the context of chronic conditions such as hypertension, successful management depends less on initial intentions and more on the psychological mechanisms that enable the translation of intention into long-term action. The temporal self-regulation theory (TST; Hall & Fong, 2007) addresses this gap by integrating motivational and self-regulatory processes underlying sustained behaviors such as medication adherence. TST offers a nuanced framework for understanding health behaviors by accounting for both motivational and volitional processes. In the motivational stage, connectedness beliefs and temporal valuations shape an individual’s intention to act. The volitional stage then considers how this intention is translated into behavior, influenced by ambient temporal contingencies such as behavioral prepotency and self-regulatory capacity. This makes TST particularly relevant for understanding medication adherence, where habitual routines, contextual triggers, and self-regulatory skills are essential for maintaining long-term compliance amid the complexities of daily life (Ajzen, 1991; Hall & Fong, 2007; Liddelow et al., 2023).

**Figure 1:**
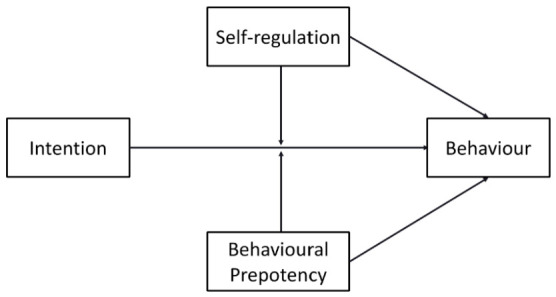
Conceptual framework for antihypertensive medication adherence using temporal self-regulation theory.

Within the TST framework, behavioral prepotency refers to the extent to which prior behavior, habitual patterns, and environmental cues increase the probability of a behavior being enacted. Behavioral prepotency not only exerts a direct effect on adherence but also moderates the intention–behavior relationship, often weakening the predictive power of intention when habits are strong or environmental cues dominate behavior (Allom et al., 2018; Booker & Mullan, 2013; Hall & Fong, 2007). Self-regulatory capacity refers to an individual’s ability to manage impulses, maintain focus, and carry out planned health behaviors, even when these behaviors require sustained effort and offer little immediate reward. In the context of medication use, self-regulation helps individuals overcome forgetfulness, manage competing demands, and remain committed to long-term treatment goals. Self-regulation may also moderate the relationship between intention and behavior. This underscores the essential role of self-regulatory capacity in supporting everyday health behaviors such as medication adherence (Allom et al., 2018; Hall, 2016; Liu et al., 2021). Building on the temporal self-regulation theory framework, the following hypotheses are proposed (Figure 1).

**H1.**  *Intention to adhere to antihypertensive medication will significantly predict medication adherence among the study population*.

**H2.**  *Behavioral prepotency and self-regulatory capacity will significantly predict medication adherence to hypertensive medication*.

**H3.**  *Behavioral prepotency and self-regulatory capacity will moderate the relationship between intention and adherence*.

Temporal self-regulation theory (TST) has demonstrated robust utility in predicting health behaviors such as alcohol consumption, diet, and physical activity (Hall & Fong, 2015; McAlpine et al., 2024). While recent applications have extended to medication adherence and self-management (Liddelow et al., 2021; Tao et al., 2024), empirical evidence remains insufficient regarding its validity in chronic disease management, where behaviors must be sustained indefinitely.

In Taiwan, hypertension remains a significant public health burden characterized by persistent non-adherence, likely exacerbated by the asymptomatic nature of the condition (Ministry of Health and Welfare, 2023). Despite the framework’s potential, current TST research often overlooks the synergistic interaction effects between intention, behavioral prepotency, and self-regulatory capacity (Liddelow et al., 2023). Neglecting these interactions limits our understanding of the boundary conditions that dictate adherence to specific diseases.

Furthermore, there is a dearth of TST-based evidence within Asian community-dwelling populations. Therefore, this study recruited community-dwelling individuals with hypertension and examined predictors of antihypertensive medication adherence using TST as the guiding framework to inform more targeted, sustainable interventions.

## 2. Materials and Methods

### 2.1. Study Design and Participants

This cross-sectional study was conducted from January to April 2024 to investigate self-reported medication adherence among adults with hypertension in Taiwan. According to the 2017–2020 National Nutrition and Health Survey, hypertension affected 26.8% of Taiwanese adults aged 18 years and older, representing an estimated 5.29 million individuals in Taiwan (Health Promotion Administration, 2023). To ensure robust statistical precision, we calculated the minimum sample size based on a 95% confidence level, a 3% margin of error, and maximum variance (*p* = 0.5), yielding a baseline requirement of 1000 participants. We then increased our final target sample to 1068 participants to provide sufficient statistical power for multivariate modeling and to detect complex interaction effects within the temporal self-regulation theory (TST) framework.

Participants were recruited through Computer-Assisted Telephone Interviewing (CATI) using a stratified random sampling design across Taiwan’s major geographic regions (Northern, Central, Southern, Eastern, and Offshore). Although our sampling plan originally included five geographic regions, only seven participants were recruited from the offshore islands. Due to this small number, these individuals were grouped with East (n = 4) and South Taiwan (n = 3) to ensure statistical reliability, leaving four regional categories in our final analysis (Table 1). To improve population coverage and minimize the exclusion of mobile-only households, both landline and mobile telephone numbers were included in the sampling frame. Trained interviewers followed standardized data collection procedures throughout the survey process. In cases of non-response, refusal, or ineligibility, the next randomly selected individual within the same geographic stratum was contacted to maintain the target sample size and regional distribution. Only fully completed interviews were retained for analysis, resulting in a final sample of 1068 adults with hypertension. No sampling weights were used in the sampling design or applied during data analysis.

Eligible participants were aged 18 years or older and reported receiving a physician diagnosis of hypertension, and those who were taking antihypertensive medication. Hypertension was defined using office blood pressure measurements, with a systolic pressure of 140 mmHg or higher, or a diastolic pressure of 90 mmHg or higher (Chiang et al., 2015). Individuals who were unable to complete the interview due to cognitive or language limitations were excluded. The study protocol was approved by the National Taiwan Normal University Institutional Review Board (Approval No. 202305HM054). All participants received a brief explanation of the study from trained interviewers and provided verbally informed consent before participating.

### 2.2. Measures

To ensure cross-cultural validity and psychometric equivalence, a standard forward–backward translation procedure was conducted prior to data collection. The original English instruments were translated into Mandarin Chinese by a bilingual researcher and independently back-translated into English by a second bilingual researcher who was blinded to the original versions. The research team reviewed and reconciled minor wording discrepancies to ensure conceptual equivalence across languages. The translated instruments demonstrated acceptable to good internal consistency reliability, with Cronbach’s α values ranging from 0.74 to 0.80. The specific instruments used are described below.

#### 2.2.1. Medication Adherence

Self-reported medication adherence behavior was measured using the 12-item Adherence to Refills and Medications Scale (ARMS), which captures both medication-taking behaviors and prescription refill practices (Kripalani et al., 2009). Participants self-reported how often they missed doses or delayed refills on a 4-point Likert scale ranging from 1 (“none of the time”) to 4 (“all of the time”), yielding a total ARMS score from 12 to 48. Because higher scores reflect more frequent non-adherence, lower total ARMS scores indicate better adherence behavior. To assess concurrent validity, participants were also administered the 4-item Morisky Medication Adherence Scale (MMAS-4), where higher scores indicate better adherence. Additionally, to evaluate criterion validity through objective behavioral data, pharmacy refill records were retrieved to calculate the Continuous Measure of Medication Gap (CMG) across three distinct observational windows: 6 months pre-study, 6 months follow-up, and 12 months post-baseline.

In this sample, the translated Mandarin version of the ARMS demonstrated good internal consistency, with a Cronbach’s alpha (*α*) of 0.75. To verify construct validity within this cohort, ARMS scores were correlated against alternative adherence metrics. To verify the psychometric and construct validity of the ARMS within this specific cohort, total ARMS scores were correlated against the alternative metrics described above. The 12-item ARMS score showed strong concurrent validity, correlating almost perfectly with its own validated short-form ARMS-7 (r = 0.950, *p* < 0.001) and sharing a strong negative correlation with the 4-item Morisky Scale (MMAS-4) (r = −0.646, *p* < 0.001). Because lower ARMS scores and higher MMAS-4 scores both reflect better adherence, this inverse relationship confirms clear structural convergence. Furthermore, criterion validity was supported by objective pharmacy refill data via the Continuous Measure of Medication Gap (CMG). The ARMS correlated significantly with CMG metrics across all analyzed timeframes: 6 months pre-study (r = 0.339), 6 months follow-up (r = 0.241), and 12 months final (r = 0.263; all *p* < 0.001). Together, these correlations confirm that self-reported ARMS scores serve as accurate indicators of true clinical behavior.

#### 2.2.2. Medication Adherence Intention

Intention was assessed using a single item: *“I intend to adhere to my medication as prescribed over the next week.”* Responses were recorded on a five-point Likert scale, with higher scores indicating stronger intention.

#### 2.2.3. Behavioral Prepotency

Behavioral prepotency was measured with a 7-item scale adapted from the Behavioral Prepotency Questionnaire. This scale captured two components: environmental cues (3 items; (Rogers et al., 2023)) and the automaticity and frequency of past behavior (4 items; (Gardner et al., 2012)). All items were rated on a 5-point Likert scale (1–5), with higher scores indicating stronger habitual tendencies and cue-driven medication-taking behavior. In the present study, the behavioral prepotency scale demonstrated excellent internal consistency (Cronbach’s *α* = 0.80).

#### 2.2.4. Self-Regulatory Capacity

Self-regulatory capacity was assessed using two related dimensions: self-control and planning ability. A culturally adapted 13-item version of the Brief Self-Control Scale (Tangney et al., 2004) was used to evaluate participants’ ability to regulate impulses and organize future actions, both of which are critical skills for maintaining consistent, long-term medication adherence. In this study, the total self-regulatory capacity scale exhibited good internal consistency (Cronbach’s *α* = 0.74).

#### 2.2.5. Covariates

Sociodemographic covariates included age, sex, geographic region of residence, and educational level. Health-related covariates included family medical history and use of chronic disease medications in addition to antihypertensive medication.

### 2.3. Statistical Analysis

We performed all statistical analyses using SAS (Version 9.4), beginning with descriptive statistics to summarize the sample characteristics. Since our primary predictors (intention, behavioral prepotency, and self-regulatory capacity) are theoretically interconnected within the TST framework, we first calculated Pearson’s correlation coefficients to evaluate their associations. To ensure the integrity of our regression models, we also conducted multicollinearity diagnostics; specifically, we calculated the Variance Inflation Factor (VIF) for each variable, where values below 5 indicated that the model was not biased by overlapping data.

To move beyond simple correlations, we utilized a hierarchical multiple linear regression to determine the unique drivers of medication adherence. This phased approach allowed us to first account for baseline differences in sociodemographic and health-related factors, such as age and comorbidities. Following this, we introduced the core TST constructs to assess their direct predictive power. In the final stage of our analysis, we incorporated two-way interaction terms to test the theory’s integrative hypotheses. This step was essential to determine whether habit strength or planning abilities significantly moderate the relationship between an individual’s intention and their actual adherence behavior. To ensure the results remained interpretable and to further reduce collinearity, all continuous variables were mean-centered prior to the creation of these interaction terms. We maintained a two-sided significance threshold of *p* < 0.05 throughout the study.

## 3. Results

Among the 1068 participants, slightly more than half were men (54.3%). The mean age was 69.4 years, with ages ranging from 34 to 97 years. Nearly half of the participants (42.8%) had attained a bachelor’s degree or higher, and just over half resided in Northern Taiwan (53.9%). A large proportion reported a family history of hypertension (74.8%), while 59.0% had at least one chronic comorbid condition and were receiving ongoing medication treatment (Table 1).

Table 2 summarizes the mean scores and Pearson correlations for the key constructs of the temporal self-regulation theory. Overall, participants reported a high level of intention to continue their medication regimen (M = 4.32, SD = 0.73). Behavioral prepotency was also high (M = 29.07, SD = 2.71), supported by consistent cues to action (M = 13.90, SD = 1.88) and a high degree of behavioral automaticity (M = 13.67, SD = 1.95). Participants demonstrated moderate to high self-regulatory capacity (M = 48.52, SD = 6.84), indicating a reasonable ability to plan, monitor, and manage their medication-taking behavior. Overall ARMS score was favorable (M = 15.89, SD = 3.53), encompassing both prescription refills (M = 5.00, SD = 1.50) and daily medication use (M = 10.90, SD = 2.62).

In line with the temporal self-regulation theory, participants who were more motivated to adhere were also more likely to have established medication-taking habits (r = 0.22, *p* < 0.001) and greater self-regulatory (r = 0.12, *p* < 0.001). Intention was negatively correlated with the adherence behavior score (r = −0.21, *p* < 0.001), indicating that stronger intention was linked to better adherence. Furthermore, behavioral prepotency (r = −0.55, *p* < 0.001) and self-regulatory capacity (r = −0.36, *p* < 0.001) were negatively associated with the ARMS score.

### Multiple Linear Regression Analysis

Before evaluating our regression models, we checked all independent variables for multicollinearity. The Variance Inflation Factors (VIF) ranged between 1.03 and 1.48, with corresponding tolerance values between 0.68 and 0.97. Because the VIF scores fall well below the conservative threshold of 5.0 and the tolerance levels sit safely above 0.10, no evidence of problematic multicollinearity was observed among the variables included in the analysis.

We then conducted a hierarchical multiple linear regression across four sequential stages to determine what predicts medication adherence. Because the ARMS is scored inversely, meaning higher scores indicate poorer adherence, a negative coefficient (B or β) points to a factor that improves adherence, while a positive coefficient highlights a barrier (Kripalani et al., 2009).

In the first model, as shown in Table 3, we entered background demographic variables, which included gender, age, education level, and family medical history. The model was statistically significant (F = 6.44, *p* < 0.001) and explained roughly 5% of the variance (R^2^ = 0.05). The results show that older age (B = −0.04, β = −0.12, *p* < 0.001) is significantly associated with better medication adherence. Conversely, higher education levels, specifically holding a high school diploma (B = 1.12, β = 0.15, *p* = 0.001) or a bachelor’s degree (B = 1.19, β = 0.17, *p* = 0.0004), were linked to poorer adherence outcomes when compared to having an elementary school education.

In the second model, we added intention, which significantly improved the model fit (ΔF = 15.87, *p* < 0.001) and accounted for an additional 5% of the variance (ΔR^2^ = 0.05; overall model F = 11.18, *p* < 0.001). Intention showed a significant negative association with the ARMS score (B = −1.02, β = −0.21, *p* < 0.001), confirming that a stronger intention to take medication directly corresponds to better real-world adherence behavior.

In the third model, we introduced the more proximal volitional constructs, which were behavioral prepotency (habit) and self-regulatory capacity. Adding these variables drastically optimized the model’s predictive power, explaining an additional 26% of the variance (ΔR^2^ = 0.26) with a highly significant step improvement (ΔF = 66.57, *p* < 0.001; overall model F = 49.97, *p* < 0.001). Consistent with our theoretical framework, both behavioral prepotency (B = −0.60, β = −0.46, *p* < 0.001) and self-regulatory capacity (B = −0.10, β = −0.19, *p* < 0.001) yielded significant negative coefficients. This suggests that stronger habitual behaviors and greater self-regulatory capacity were associated with better adherence outcomes.

In the fourth model, we added the interaction terms (Intention × Behavioral Prepotency and Intention × Self-Regulatory Capacity). Although the overall model remained significant (F = 42.78, *p* < 0.001), these specific interaction effects were not statistically significant (ΔR^2^ = 0.00, ΔF = 0.21, *p* > 0.05). The absence of significant interaction effects indicates that behavioral prepotency and self-regulatory capacity were associated with adherence independently of intention, rather than showing evidence of moderating the relationship between intention and adherence.

## 4. Discussion

This study applied the temporal self-regulation theory (TST) to examine how behavioral and psychological factors shape hypertension medication adherence among community-dwelling adults in Taiwan. After adjusting for demographic variables, the combined effects of intention, behavioral prepotency, and self-regulatory capacity explained 36% of the variance in adherence (R^2^ = 0.36, *p* < 0.0001). This explanatory power exceeds that of several prior TST applications (Evans et al., 2017; McAlpine et al., 2024), although it is slightly lower than studies examining supplement or medication use (Allom et al., 2018; Liddelow et al., 2021).

Study findings provide insights into the persistent challenge known as the intention–behavior gap in chronic illness management. Medication adherence was supported not only by motivation but also by the extent to which medication-taking had become embedded in daily routines through habitual processes and self-regulatory capacity (Liddelow et al., 2023). Despite strong intentions, the actual ARMS score was only moderate, reflecting the well-documented intention–behavior gap commonly observed in chronic disease management (Sheeran & Webb, 2016). Such mechanisms have been increasingly recognized across conditions requiring long-term self-care (Liddelow et al., 2020a, 2023).

In this study, although intention remained a significant predictor of medication adherence (B = −0.40, β = −0.08, *p* = 0.001), behavioral prepotency (β = −0.46, *p* < 0.001) and self-regulatory capacity (β = −0.19, *p* < 0.001) showed stronger associations with adherence in the model. Participants with consistent medication routines, such as taking medication at the same time daily, using pill organizers, or integrating medication use into existing habits, demonstrated markedly better adherence. These automatic routines reduced reliance on conscious intention and offered resilience during stressful or busy periods. This pattern also aligns with the Health Action Process Approach, which proposes that behaviors gradually shift from intentional to automatic during maintenance phases (Allom et al., 2018; Booker & Mullan, 2013; Mohammadi et al., 2025; Schwarzer & Luszczynska, 2008). Barriers such as forgetfulness, irregular schedules, or uncertainty about medication necessity could impede habit formation, whereas stable routines, external cues, and family encouragement supported automatic behavior (Badawy et al., 2020; Chang et al., 2021; Dupclay et al., 2012; Ghai et al., 2024; Jimmy & Jose, 2011). In terms of the effects of self-regulatory capacity, individuals with stronger self-control, better planning, and greater attentional regulation were more capable of sustaining their regimen despite stress, competing demands, or immediate discomfort. These individuals generally maintained healthier behaviors across domains, including nutrition, physical activity, and impulse control, all of which require sustained effort for long-term gains (Allom et al., 2018; Hagger & Hamilton, 2024; Hall, 2016; World Health Organization, 2024).

Importantly, the findings suggest that behavioral prepotency and self-regulatory capacity were associated with adherence, with no evidence that either construct significantly moderated the intention–adherence relationship. These findings support that two independent mechanisms are at work: habit reduces cognitive effort, while self-regulation helps individuals manage disruptions and remain focused over time (Hagger & Hamilton, 2024; Liddelow et al., 2023). Effective hypertension management, therefore, depends not only on motivation but also on the routines and self-regulatory skills that make adherence sustainable.

The demographic characteristics provide additional context for medication adherence. Older age was initially associated with better medication adherence in Model 1 (β = −0.12, *p* < 0.001). However, this association weakened considerably after the psychological variables were included in Model 3 (β = −0.05, *p* = 0.04). This finding suggests that the association between age and adherence may be partly accounted for by the inclusion of habitual and self-regulatory variables in the model. Other studies also show that these factors naturally help people form habits and maintain consistent medication-taking over time (Ge et al., 2023; Jin et al., 2008). Education showed a counterintuitive effect, with participants holding high school or bachelor’s degrees adhering slightly less than those with elementary-level education. Consistent with previous findings (Jia et al., 2022), education alone does not ensure better adherence, emphasizing the need for interventions that consider lifestyle, cognitive factors, and decision-making patterns among more educated adults.

### 4.1. Strengths

This study’s nationally representative sample supports strong generalizability, while the telephone-based data collection minimized missingness. As one of the few studies applying TST to hypertension medication adherence, it offers novel insights into how intention, habit strength, and self-regulatory capacity collectively shape daily treatment behaviors. These mechanisms are relevant for other chronic conditions requiring lifelong adherence.

### 4.2. Limitations

A cross-sectional design restricts causal inference; longitudinal or experimental designs are needed to establish temporal ordering. Reliance on self-reported adherence introduces recall and social desirability bias. Future research may incorporate objective data sources such as pharmacy refill records or digital monitoring to enhance accuracy. Although the CATI sampling and recruitment procedures strictly followed a rigorous, scientific protocol, detailed call records from the third-party contractor were not available. Therefore, the response rate, number of unsuccessful contact attempts, and formal assessment of non-response bias could not be reported.

## 5. Conclusions

This study demonstrates that although intention is important for hypertension medication adherence, behavioral prepotency and self-regulatory capacity are also the drivers of consistent behavior. Interventions that help individuals with hypertension build structured routines, use environmental cues, and strengthen self-regulation are likely to be the most effective for supporting long-term adherence. Hypertension management, as well as chronic illness care more broadly, is therefore not only a medical issue but also a behavioral one. Integrating motivational strategies with approaches that reinforce habitual routines and self-regulation can help individuals translate their intentions into actual medication use, ultimately improving long-term health outcomes and supporting lifelong chronic disease management.

### Implications

Effective adherence support may target the core mechanisms of the temporal self-regulation theory by:

- Support routine and habit formation: Help individuals with hypertension anchor medication-taking to daily cues, encourage the use of pill organizers, reminders, or digital tools, and simplify regimens for those with lower self-regulatory capacity.
- Strengthen self-regulatory skills: Provide planning, goal setting, and time-management strategies to help individuals with hypertension maintain adherence despite competing demands, disruptions, or stress.
- Future research: Longitudinal and experimental designs are needed to examine how habits and self-regulatory capacity evolve over time, identify when self-regulatory resources decline, and determine the optimal timing for booster interventions.

## Figures and Tables

**Table 1:** Demographics and clinical characteristic variables (N = 1068).

Variable	Frequency/Mean	Percent/SD
**Gender**		
Male	577	54.03%
Female	491	45.97%
**Age**	69.42	10.15
**Residency Area**		
North Taiwan	576	53.93%
Central Taiwan	207	19.38%
South Taiwan	253	23.69%
East Taiwan	32	3%
**Educational level**		
Elementary School	164	15.36%
Junior High School	127	11.89%
High School	320	29.96%
Bachelor’s Degree	457	42.8%
**Family Medical History**		
No	269	25.19%
Yes	799	74.81%
**Chronic Disease Drug Use**		
No	438	41.01%
Yes	630	58.99%

Note: Offshore (n = 7) comprises East Taiwan (n = 4) and South Taiwan (Penghu County, n = 3).

**Table 2:** Descriptive statistics and correlations for the main variables based on the TST (N = 1068).

Main Variables	Mean	SD	1	2	3	4
1. Intention	4.32	0.73	1	0.22 ***	0.12 ***	−0.21 ***
2. Behavioral prepotency	29.07	2.71		1	0.3 ***	−0.55 ***
3. Self-regulatory Capacity	48.52	6.84			1	−0.36 ***
4. Medication adherence	15.89	3.53				1

*** *p*-value < 0.001.

**Table 3:** Regression analysis of background variables, intention, behavioral prepotency, and self-regulatory capacity on behaviors (N = 1068).

Predictor	B [95% CI]	β	*p*-Value	R^2^	ΔR^2^	F
**Model 1**			**<0.001**	**0.05**	**0.05**	**6.44** *
Female (ref: Male)	−0.26 [−0.69, 0.17]	−0.04	0.23			
Age	−0.04 [−0.06, −0.02]	−0.12	<0.001			
Junior High School (ref: Elementary)	−0.03 [−0.84, 0.78]	−0.002	0.95			
High School	1.12 [0.45, 1.80]	0.15	0.001			
Bachelor’s Degree	1.19 [0.53, 1.86]	0.17	0.0004			
Family Medical History (Yes)	−0.10 [−0.58, 0.39]	−0.01	0.70			
**Model 2**			**<0.001**	**0.10**	**0.05**	**11.18** *
Intention	−1.02 [−1.29, −0.74]	−0.21	<0.001			
**Model 3**			**<0.001**	**0.36**	**0.26**	**49.97** *
Female (ref: Male)	−0.03 [−0.38, 0.33]	−0.004	0.89			
Age	−0.02 [−0.04, −0.001]	−0.05	0.04			
Intention	−0.40 [−0.64, −0.16]	−0.08	0.001			
Behavioral Prepotency	−0.60 [−0.66, −0.53]	−0.46	<0.001			
Self-Regulatory Capacity	−0.10 [−0.13, −0.07]	−0.19	<0.001			
**Model 4**			**<0.001**	**0.36**	**0.00**	**42.78** *
Intention × Behavioral Prepotency	−0.002 [−0.08, 0.08]	−0.01	0.96			
Intention × Self-Regulatory Capacity	−0.007 [−0.04, 0.03]	−0.10	0.68			

Note: Lower scores on the Adherence to Refills and Medications Scale (ARMS) reflect superior adherence. Consequently, negative coefficients indicate factors that promote adherence behavior. * Indicates that the overall regression model is statistically significant (*p* < 0.001).

## Data Availability

The datasets generated and analyzed during the current study are available from the corresponding author upon reasonable request.

## Abbreviations

The following abbreviations are used in this manuscript:

ARMS	Adherence to Refills and Medications Scale
CATI	Computer-Assisted Telephone Interviewing
CVD	Cardiovascular Disease
TST	Temporal Self-Regulation Theory
TPB	Theory of Planned Behavior
HHD	Hypertensive Heart Disease
DALYs	Disability-Adjusted Life Years
WHO	World Health Organization
SAS	Statistical Analysis System
SD	Standard Deviation
